# Microscopy of the Teeth, Continued

**Published:** 1868-05

**Authors:** S. P. Cutler

**Affiliations:** Professor of Chemistry and Microscopy in the Ohio Dental College, Cincinnati. Formerly Professor of Chemistry and Natural Sciences in the Botanic Medical College, Memphis, Tenn.


### MICROSCOPY OF THE TEETH—CONTINUED.

BY S. P. CUTLER, M. D., A. E. G., D. D. S.

Professor of Chemistry and Microscopy in the Ohio Dental College, Cincinnati. Formerly Professor of Chemistry and Natural Sciences in the Botanic Medical College, Memphis, Tenn.

FALLACIES must supplant truth and truth must perish, because certain men will it to be so. A sweeping article in the May number, 1868, of the *Dental Cosmos*, commencing on page 246, sets out with the broad assertion that at least the writers in the Dental profession, so far as relates to the Dental structures, amount absolutely to nothing, or less than nothing; in other words, ignoring altogether all that has heretofore been written. As a matter of course, I happen to be included in that category as well as others. But the writer or rather speaker, is going to tell us all about it; something new, so it is, and we shall see what that something is. After ignoring both the dentinal nerve fibrils and liquor sanguinis, as tubular occupants, he gives us a *tertium quid*, or a third something which is proclaimed as eureka, the golden egg, philosopher's stone, or something else. The learned body to whom these remarks were made, voted thanks—for calling them all fools. (See article referred to.)

Again, on same page says,—First, as to the soft solid, fibril-like extension of matter called dentinal fibrils, which proceed inward from the pulp and fill the dentinal tubules, which are, truly speaking, canals. (Says,) Those have been imagined to be nerve fibres, and we have frequently seen the assertion made that those nerve fibres have been demonstrated (*a hint at myself*). (Says,) I cannot persuade myself that whoever makes this claim, has ever seen a single nerve fibre under the microscope. Says more in relation to nerve bundles, leashes or trunks. Same page says, The finest tubuli is $\frac{1}{8,000}$ or $\frac{1}{10,000}$ of an inch, and that nerve fibres can be seen as fine as the $\frac{1}{50,000}$ or $\frac{1}{60,000}$ of an inch. Now, let us see. Carpenter estimates the diameter of the tubuli at the pulp cavity at $\frac{1}{10,000}$ of an inch. Now, follow out the branching of the tubuli, and it will be discovered that the coronal branches are not more than the $\frac{1}{60,000}$ of an inch in caliber. Now what has he found. In the first place, next page says, these fibrils of nerves have never been traced into the pulp, that is, he never has seen them so traced. He has found that instead of a nerve fibril, that there is a fibril of connective tissue occupying the tubuli and connecting with the pulp; same page says, In the absence of any traces in the dentine of any tissue resembling a nerve fibre, we may imagine the dentinal fibrils to embody the function of sensibility for the tooth; but we are not left to imagine that they are nerve fibres, since it has been demonstrated that they are extensions of connective tissues; then gave some specimens to illustrate his remarks. Now, he wishes it understood, that this connective tissue performs in this instance a vicarious function, that of conveying sensibility from the dentine to the pulp connective tissue, and in some way, which is not explained, and from this tissue to the nerves in the pulp, and from there to the brain through the trigemina.

He does not pretend to inform the reader how these sentient impressions are carried from the diseased dentine to the nerve of the pulp, nor how there impressed. He further regards it as a vaso-motor or nutrient, and not a sentient nerve. I would here ask, what causes pain if the nerve can not take any cognizance of painful impressions.

He ignores with one sweeping phrase all that I have written concerning the connection of nerve fibrils from the central plexus of the pulp through the pulp membrane, and through the tubuli to the cement and enamel, simply because he has not been able to trace them out—denies the whole thing. This is rather strong language.

How does he attempt to prove his connective tissue theory? Simply by saying that these dentinal fibrils resemble that kind of tissue, and not ordinary nerve fibril, of axis, cylinder and neurilemma (*and ought also to have said neurine*), as being true nerve structure.

He does not allow the teeth to be an exception to other organs only in one respect, that is, that the dentine does not, after it is once built up, receive nutrition nor undergo waste; in other words, dentine is dead tissue. Now, here is dead tissue, with connective tissue fibrils conveying sensation to a motor or nutrient nerve (*dental*). What congruity is there in such arguments? simply, none at all; if so, I can't see it. Now this last dogma is more untenable, if any odds, than the fluid theory of nerve sensation, which I shall discuss in some subsequent article, where I shall endeavor to show the fallacy of that doctrine. I cannot allow the teeth which are so intensely sensitive to morbid impressions, to give so much trouble and suffering when diseased, and when operated upon, to go on and give all the trouble we see, without allowing them at least in common with other organs of the body, to have nerves, and millions of them at that; say, from ten to twenty or more millions of them in the dentine. Now again, if connective tissue can convey sentient impressions and have a fibrillous structure, why not at once admit them to be nerves, even if they are somewhat different in minute structure from some other nerve filaments. I take it that the teeth are peculiar to themselves, and have no homologue in the economy in scarcely any respect, and have a peculiar office to perform, differing from any other set of organs in many respects.

Does the gentleman claim that the terminal branches of all nerve fibres are under all circumstances precisely of the same structure, say of the five senses? Does he pretend to say that the terminal filaments of the nerves of touch, smell, taste, seeing and hearing, are precisely alike, when the impressions received are so widely different that gives these different functions? Take the retina and the cochleal distribution of the portio mollis, and examine and see if their structures are precisely the same in all respects. Has this point been settled yet? Again, if all nerve fibres were anatomically precisely alike, how could there be different phenomena manifested as in the case of the special senses, that of general sensation and motion, also nutrition? These are still unsettled and mooted points, though it seems to me there is and must be a marked difference, though I do not claim to be an authority on these points, only so far as the teeth are concerned. On page 250, the writer denies the existence of capillaries in the pulp, and maintains that the arteries terminate in veins, and hence there is no nutrition carried on even in the soft pulp, which is made up of alveolar or connective tissue, and the blood-vessels and system, or plexuses of nerves, which have been minutely described by myself in the *Cosmos*. Can any person show where arteries terminate abruptly in veins? If so, what is the use or object of such circulation. On same page, he denies that dentine after once built up, undergoes any change of either nutrition or waste, only decay of teeth. This I believe is in opposition to all authority, also without reason or facts to sustain it. Here he regards the dentine as dead matter, as all tissues are dead where there is no nutrition and waste; still he maintains that the dentinal fibrils of connective tissues in some way do convey sensation; yet still denies these fibrils the right of nutrition. On same page he says, no liquor sanguinis, but only water and salts are found outside of the pulp membrane. I would here ask, who has analyzed this fluid to so determine, or is this theory, without demonstration to sustain it? Now if this be true, what use is there of any such fluids at all, or the salts? He does not even give the kind of salts; there are a great many kinds of salts in the fluids of the body for various uses. I would ask if these connective tissue fibres could be nourished with water and salts alone? Though they are not nourished, and consequently it is immaterial. We must then infer that these arteries and veins exude this water and salts through their walls. This again is a new feature in physiology.

Right here again, I take issue and deny this doctrine. I regard the whole thing as based on speculative theory alone, without any foundation in facts based upon actual observation and demonstration. I should not come out thus plain and pointed, had the writer not licensed me to do so. I also deem this subject of too much importance to pass it lightly over, when such untenable views are advanced and listened to with so much credence by so many intelligent members of the profession.

Let me now examine a little into the nature and character of areolar tissue or connective tissue, sometimes called the reticulated connective tissue; so called by Kolliker, the fibro-cellular by Hassall; and areolar tissue by Todd and Bowman. This last is regarded by Peasley as the most appropriate. Kolliker includes under the term connective tissue, the white fibrous elastic fibres, fat cells, cartilage cells, and pigment cells of different kinds. Lehman regards connective and areolar tissue as the same. Connective tissue is that tissue that connects parts and organs, and is composed of white fibrous or cellaginous and yellow fibrous blended together. No nerves or blood-vessels are found in these tissues, and no other vital properties save that of securing and maintaining their development; but they possess physical properties merely, such as great strength and flexibility, and inextensibility. They contain a large amount of water on which their flexibility depends. Tendons, articular ligaments and aponeurosis, are made up mostly of the white fibrous tissue; it also forms a large portion of fibrous membranes, as the periosteum, perichondrium, dura mater, and, with the yellow fibrous, forms areolar tissue. Need I say more about this tissue. We have seen that these tissues are the least vital of any, simply forming connecting links in the economy, and are not for the purpose of carrying or conveying the most acute of all nervous impressions, such as the sensibility of a decayed tooth of a certain character. Let me ask right here, are the intelligent Dentists prepared to adopt all that has been advanced by the gentleman, and ignore all that they had hitherto learnt from their reading and experience in Dental operations? I imagine not. I am writing this article in self defence, and that of the profession at large; and expect to maintain what I have written until some more reasonable and convincing deductions are advanced, when I shall be ready at once to change my views, and not until then. Again, because the gentleman or any one else has not been able to successfully prepare specimens so as to show the nerve connections from dentine, to and through the pulp, does not amount to any thing more than negative proof, which amounts to nothing in science. I am able to demonstrate all I have asserted. Again, do ligaments, cartilages, and other similar connective tissue possess acute sensibility like the teeth? It has been shown that they do not.

[TO BE CONTINUED.]

